# Co‐targeting of lysosome and mitophagy in cancer stem cells with chloroquine analogues and antibiotics

**DOI:** 10.1111/jcmm.15879

**Published:** 2020-09-15

**Authors:** Md. Abdul Alim Al‐Bari

**Affiliations:** ^1^ Department of Pharmacy University of Rajshahi Rajshahi Bangladesh

**Keywords:** antibiotics, autophagy, chloroquine analogues, CSCs therapy, drug repurposing, mitochondrial target

## Abstract

The catabolic autophagy eliminates cytoplasmic components and organelles via lysosomes. Non‐selective bulk autophagy and selective autophagy (mitophagy) are linked in intracellular homeostasis both normal and cancer cells. Autophagy has complex and paradoxical dual role in cancers; it can play either tumour suppressor or tumour promoter depending on the tumour type, stage, microenvironment and genetic context. Cancer stem cells (CSCs) cause tumour recurrence and promote resistant to therapy for driving poor clinical consequences. Thus, new healing strategies are urgently needed to annihilate and eradicate CSCs. As chloroquine (CQ) analogues show positive clinical outcome in several clinical trials either standalone or combination with several chemotherapies. Moreover, CQ analogues are known to eliminate CSCs via altering DNA methylation. However, several obstacles such as higher concentrations and dose‐dependent toxicity are noticeable in the treatment of cancers. As tumour cells predominantly rely on mitochondrial actions, mitochondrial targeting FDA‐approved antibiotics are reported to effectively eradicate CSCs alone or combination with chemotherapy. However, antibiotics cause metabolic glycolytic shift in cancer cells for survival and repopulation. This review will provide a sketch of the inhibiting roles of current chloroquine analogues and antibiotic combination in CSC autophagy process and discuss the possibility that pre‐clinical and clinical potential therapeutic strategy for anticancer therapy.

**Lists of main topics**

- Autophagy is an emerging potential therapeutic target for multiple disorders including multiple malignant tumours. Autophagy has both suppression role in tumour initiation and promotion action in tumour progression, and this controversy role of autophagy has led to dilemma over whether or how targeting of autophagy therapeutically should be undertaken for efficient treatment of cancers.
- Chloroquine analogues are established autophagy inhibitors from malaria treatment. When lysosomotropic action of chloroquine analogues is elucidated, these drugs have become popular for autophagy suppressors.
- The clinical studies for chloroquine analogues are executed owing to their prior Food and Drug Administration (FDA) approval and expanded indication in the treatment of inflammatory diseases.
- Although pharmacokinetic parameters and safety profiles of chloroquine analogues are less favourable in cancer patients, combination treatment data are emerging.
- Maintenance of cancer stem cells (CSCs) is associated with the endosome/lysosome pathway, and propagation and clonal expansion of CSCs are dependent on mitophagy. Thus, the pharmacological inhibition of lysosomal flux and mitochondrial biogenesis may effectively block CSCs.

## 1 INTRODUCTION

Cancer becomes the principal originator of morbidity and mortality rates in the next few years in both developed and transitioning economy countries.^1^, ^2^, ^3^ The scientific society has given enormous struggles and endeavour to advance innovative strategies for cancer therapy to deal efficiently with this uprising and complicating issue. Although the advanced cancer therapies are progressing from the survival rate of patients, cancer still persists one of the most fatal epidemic maladies. Cancer recurrence and metastatic progression are frequent in patients receiving conventional chemotherapy or radiotherapy.^4^ As contemporary chemotherapeutics are most efficient for eliminating very quickly propagating cells, the failure rate of conventional therapies is likely associated with a relatively rare slowly proliferating culture of cancer cells stay in tumour, called cancer stem cells (CSCs).^5^, ^6^ CSCs have been exhibited to be resistant to traditional chemotherapy as well as radiation.^7^ Residual memory CSCs disappeared after clinical treatment are suggested responsible for the re‐survival of tumours and for their progressive metastasis.^8^ It has also been suggested that the most metabolic active CSCs have heightened biogenetic rate of mitochondria as compared to normal cell correspondents.^9^, ^10^ Thus, a great attempt has been concerned to the new drug development that is capable to correctly target biogenesis of mitochondria‐associated CSCs.

The conventional drug discovery and development process are an indeed challenging field in terms of rising and unsustainable costs, and time‐exhausting tasks, with a high frequency of failure rate.^11^ Thus, pharmaceutical companies have decided to decrease annual investment regarding classical drug discovery^12^ and healthcare systems have faced the substantial challenge in their survival for commercial sustainability inflamed by paying of prescription drugs.^13^ In this context, drug repurposing (new therapeutic uses or indications are found for existing drugs) appears as a new platform for the pharmaceutical industries, patients and healthcare payers.^14^, ^15^ Moreover, drug repositioning (also called drug repurposing) approach may conquer many tremendous obstacles involved in new drugs discovery because of having established pharmacokinetics, pharmacodynamics and toxicity profiles, approval by several regulatory agencies FDA (US) and EMEA (Europe), and these recognitions accelerate the assessment of the agents in clinical trials.^16^, ^17^ Furthermore, drug repurposing may discover novel molecular regulatory pathways involved in cancer regrowth or admit new molecular targets for cancer therapy.^18^ It has been exemplified that repurposing drugs chloroquine (CQ) analogues and antibiotics are known to accelerate the therapeutic capacity of chemotherapy by eliminating CSC traits of invasive progression in tumours.^19^, ^20^ Thus, repurposing drugs play an important role to eradicate CSC‐mediated tumorigenesis.

## 2 ROLE OF BULK AUTOPHAGY IN CANCER

Macroautophagy (hereafter referred to as autophagy) is an evolutionarily conserved, cellular homeostatic process that facilitates nutrient recycling via lysosomal degradation of potentially harmful cytoplasmic entities.^21^, ^22^ It has been widely established that the bipolar nature of autophagy exists in cancers.^23^, ^24^ Autophagy act as either tumour suppressor or tumour promoter depending on tumour type, stage of tumour development, tumour microenvironment and genetic context.^25^, ^26^ Although autophagy limits cancer development in the early stages of tumorigenesis, it can also have a pro‐tumoral role in more advanced cancers, promoting primary tumour growth and metastatic spread.^25^ Under normal conditions, cells utilize basal levels of autophagy to aid in the maintenance of biological function, homeostasis, quality control of cell contents and elimination of old proteins and damaged organelles.^27^ Additionally, autophagy in stem cells is related to the maintenance of their unique properties, including differentiation and self‐renewal.^28^, ^29^ However, many established malignant cells have high levels of basal autophagy even in fed conditions.^30^, ^31^ In contrast, autophagy in normal cells generally occurs at low levels and is only up‐regulated in response to stressful conditions such as starvation. Moreover, some anticancer drugs can regulate autophagy. Therefore, autophagy‐regulated chemotherapy can be involved in cancer‐cell survival or death.^32^ Additionally, the regulation of autophagy contributes to the expression of tumour suppressor proteins or oncogenes. Tumour suppressor factors are negatively regulated by mechanistic target of rapamycin (mTOR) resulting in the induction of autophagy and suppression of the cancer initiation.^33^ In contrast, oncogenes may be activated by mTOR, class I PI3K (phosphoinositide 3‐kinases) and AKT (also known as protein kinase B), resulting in the suppression of autophagy and enhancement of cancer formation.^25^, ^34^ Although these dual‐complex mechanisms make autophagy a challenging target for anticancer therapeutics, a better understanding of the autophagic roles in different stages of tumorigenesis, specific cellular and extracellular context and the crosstalk between autophagy and apoptosis should all be taken into consideration to better harness autophagy in cancer treatment.^35^

Interestingly, an intricate link between autophagy and cancer is established when *Beclin 1* (*BECN1*), an essential autophagy gene, is found to suppress breast tumorigenesis.^36^, ^37^ In several cancer‐cell lines and mice models, the loss of *BECN1* results in an inhibition of autophagy and an upsurge in cell proliferation.^36^, ^38^, ^39^ In addition, the *BECN1* gene is monoallellicaly deleted in 40%–75% of breast, ovarian and prostate cancers.^36^, ^40^, ^41^ It is also found that the overexpression of *Beclin 1* can inhibit the growth of colon cancer cells,^42^ nasopharyngeal carcinoma^43^ and CaSki cervical cancer cells.^44^ Due to the genomic close proximity of the *BRCA1* (breast cancer 1, early‐onset gene) and the *BECN1* gene at the 17q21 chromosome, it was assumed that *BECN1* deletions are rather a passenger event.^45^ Tumour suppressor gene deletions require additional modulators to form cancer. In human breast and ovarian cancers, *BECN1* is often co‐deleted with *BRCA1*. This led to the hypothesis that *BECN1* loss is a passenger event and is only deleted due to its proximity to *BRCA1*.^45^, ^46^ *BRCA1* is frequently mutated in familial cases of breast and ovarian cancer, being relatively rare in sporadic cancers, and it is a classical tumour suppressor, as only one copy is sufficient to maintain its function. By contrast, the loss of just one allele of *BECN1* is sufficient to induce tumorigenesis,^38^, ^39^ and therefore, it is suggested as a haploinsufficient tumour suppressor. Furthermore, two survival analyses on the TCGA (Cancer Genome Atlas Project) and METABRIC (Molecular Taxonomy of Breast Cancer International Consortium) data set showed that a worse survival probability was associated with the lower *BECN1* but not with the *BRCA1* mRNA expression in all breast cancer types,^47^ indicating that in sporadic breast cancers, *BECN1* is a driver rather than a passenger event.

Autophagy also maintains cancer‐cell re‐survival during metabolic stressful conditions, and these mediate resistance to therapies such as chemotherapies or radiation.^48^, ^49^ Thus, induction of autophagy in cancer cells is associated with stress tolerance mechanism when these cells are experienced to nutrient starvation, hypoxic conditions or anticancer therapies.^50^, ^51^, ^52^ In well‐established tumours, the stress‐induced autophagy allows tumour cell regrowth which in turn expedite tumour cell advancement and negotiate resistance to anticancer therapies.^53^ As a result, inhibiting pro‐survival (cytoprotective) autophagy in cancer cells has been shown to augment the effectiveness of anticancer therapy by promoting apoptotic cell death.^54^, ^55^ Although these dual‐complex mechanisms make autophagy a challenging target for anticancer therapeutics, a better understanding of the autophagic roles in stages of tumorigenesis, specific cellular and extracellular context and the crosstalk between autophagy and apoptosis should all be taken into consideration to better harness autophagy in cancer treatment. Although autophagy modulation has promised as an emerging therapeutic strategy for certain cancer types, major challenges remain unclear. For examples, higher chemotherapy doses may cause toxic side effects and it is contradiction whether autophagy‐modulating agents may significantly affect the tumour cells. Furthermore, there is doubt existence about an actual tissue‐derived autophagy measurement, especially inaccessible in solid tumours.^18^, ^56^ Therefore, a better intervention of chemotherapeutic combination is required for modulation of inherent autophagy properties. Thus, treatment strategies of cancers that modulate autophagy both inducing and inhibiting concomitantly emphasize a better understanding for improved therapeutic outcome.

### 2.1 Targeting lysosome in autophagy by chloroquine analogues

Chloroquine (CQ) analogues such as hydroxychloroquine (HCQ), quinacrine (QN), mefloquine (MQ), Lys05, verteporfin, clioquinol SAR405, spautin‐1 (***s***pecific and ***p***otent ***aut*** phagy ***in***hibitor *1)*, ARN5187, VATG (Van Andel‐T‐Gen)‐027 and VATG‐032 and its other derivatives are well‐known repurposing success stories because these analogues are effective, inexpensive, well‐tolerated in humans.^57^, ^58^ CQ analogues, for example HCQ, MQ and verteporfin, are FDA‐approved agents generally applied for the treatment of malaria, systemic lupus erythematosus, rheumatoid arthritis and photodynamic therapy, but their potentials as anticancer agents have currently appeared.^57^ As lysosomotropic agents, CQ analogues efficiently deacidify lysosomal lumens by changing permeability of lysosomal membrane potential (LMP).^59^ Accumulating lines of evidence suggest that CQ analogues favourably induce apoptosis and necrosis in cancer cells such as breast cancer, colon cancer, glioma and glioblastoma compared with normal cells either in standalone or in combinations with chemotherapy.^53^, ^60^ In the context, it has been found that CQ analogues have direct actions on diverse kinds of cancers that influence chemotherapeutic actions, for example inhibition of both multidrug resistance pump and autophagy, intercalation in DNA and improving the penetration of chemotherapies in cancer cells or solid tumour tissues.^61^, ^62^ In these cases, the lysosome‐deacidifying property of CQ analogues seems the most vital parameter for improving efficacy and specificity for cancer therapies.

**Figure 1:**
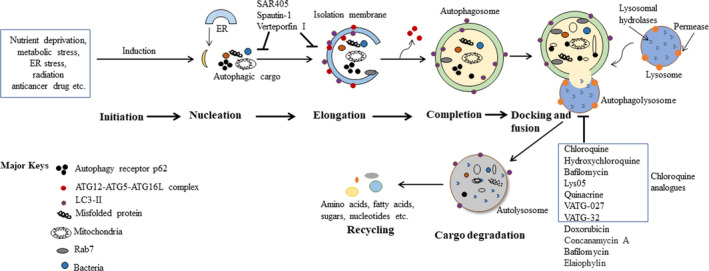
An overview of mammalian autophagy process. Starvation, growth factor deprivation, low energy and hypoxia are well‐established autophagy (specifically, macroautophagy) inducers. These culminate in mTORC1 inhibition and AMPK (5' AMP‐activated protein kinase) activation, which, in turn, positively regulate the UNC51‐like kinase 1 (ULK1) complex through a series of phosphorylation events. Induction of the ULK1 complex subsequently activates the class III PI3K complex, which leads to PI3P (phosphatidylinositol 3‐phosphate) synthesis in isolation membranes (IMs) and initiates autophagy. Numerous molecular events are subsequently activated in the autophagy pathway, including initiation, nucleation, elongation, autophagosome maturation and cargo degradation. The IMs appear to have several sources, such as the ER membrane, Golgi apparatus and trans‐Golgi network, plasma membrane, endosomal compartment and mitochondria. The two ubiquitin‐like conjugation systems **A**u**T**opha**G**y‐related 12 (ATG12)‐ATG5‐ATG16L1 complex and LC3 (microtubule‐associated proteins 1A/1B light chain 3B)‐II participate after their activation in the expansion of the double membrane and the closure of the isolation membrane. Once it is completed, the structure is called an autophagosome. After elongation and closure, the newly formed autophagosome may fuse with a late endosome to form an amphisome, or it may fuse directly with a lysosome to form an autolysosome, allowing the degradation of autophagic substrates. Once the cargos are degraded, the product macromolecules are exported to the cytosol to be recycled by the cell for ATP production and biosynthesis

CQ analogues also sensitize triple‐negative breast cancer (TNBC) cells, categorized by a plenty of chemotherapy‐resistant breast cancer stem cells (CSCs) as well as chemotherapy‐resistant pancreatic CSCs to where CQ analogues efficiently prevent autophagy.^62^, ^63^, ^64^ Thus, CQ analogues need to be more discovered in the scientific background as their victory may benefit to further quickly progress the poor diagnosis of patients with TNBC or pancreatic cancer. Interestingly, recent evidence suggests that HCQ in combination treatment with mTOR inhibitors such as temsirolimus significantly suppresses tumour growth in vitro and in vivo.^65^, ^66^ Here the period of treatment and acceptable dose of HCQ differentially affect medical profits (best outcome achieved with 1200 mg HCQ twice daily). Another clinical trial (phase 1 study) HCQ (600 mg) in blending with temozolomide (TMZ) indicates suppression of autophagy in humans. However, an increased dose of HCQ is indispensable for noticeable clinical outcome.^67^ Moreover, CQ also potentiates the cytotoxic effect of TMZ by inhibiting mitophagy in glioma cells.^68^ These reports strongly suggest that CQ analogue in combination with other autophagy‐modulating agents may significantly improve cancer treatment regimen if robust and rapid treatment strategies are necessary. More importantly, there are numerous concurrent clinical trials assessing CQ analogues in combination with chemotherapies in patients with multiple cancers.^53^ However, there are many highly debatable questions remaining as these analogues denote the most efficient agents for suppressing autophagy. For example, (a) the analogues may be required higher concentrations (µM levels) to attain adequate inhibition of autophagy in vitro and in vivo which is inconsistently achievable in humans than conventionally used for malaria and rheumatic disorders.^69^ Accordingly, HCQ combination with chemotherapeutic agents, proteasomal inhibitors, mTOR inhibitors and/ or radiation therapy has been revealed to outcome in little response rates in the initial clinical trials. Furthermore, the higher doses of HCQ used in clinical trials produce significant interpatient variability of autophagy inhibition. In addition, the half‐lives of the analogues account for long times (eg 22.4 days for HCQ), which account for the chronic side effects, including retinopathy.^59^ As sustained autophagy induction and autophagy addiction are inimitable to cancer cells, supposedly long‐term autophagy inhibition can provide a healing window to favourably affect cancer cells. However, higher maintenance of HCQ dose in a cancer patient will unavoidably affect normal cells too; (b) CQ analogue unable to suppress autophagy in acidic extracellular microenvironment (pH 6.5) in solid tumour due to reduced cellular uptake of the agents^70^; (c) some clinical trials have revealed dose‐dependent toxicities such as neutropaenia, thrombocytopaenia and sepsis when HCQ is given in combination therapy^71^; and (d) finally, CQ‐associated chemo‐sensitization to chemotherapy seems to be an autophagy‐independent occurrence.^72^ These data strongly support a necessity to investigate better therapeutic strategy with specific molecular mechanism in modulating of autophagy in cancers. Further research will be required to identify and develop for additional effective and acceptable CQ analogues as autophagy suppressors, as well as outline the prime dose and dose interval that leads to highest the therapeutic activity during cancer therapy. However, the successful drug repositioning approach has primarily been by serendipitous discovery or clinical observation, such as the rich history and serendipitous indications for chloroquine^59^ (**Table** **1** and **Figure** **1**). Thus, scientists from the repurposing drugs in oncology (ReDO) project highlighted the potentiality of CQ analogues for cancer treatment by acting on both the cancer cellular level and the tumour niches and suggested that these analogues could propose important clinical advantages for cancer patients, particularly in combination with conventional anticancer treatments.

**Table 1:** Key serendipitous events in the history of CQ analogue development that led to the successful targeting of autophagy in cancer

Year	Major discovery/events
Before 1532	Quina‐quina bark is indigenously used in South America to treat febrile illness
1632	Quina‐quina bark is used to treat for 'tertian fever' in Peru; Jesuit priest Bernabe’ de Cobo transported from Peru to Europe (Spain)
1629‐1633	The Romantic legend of Countess of Chinchon cured with quina‐quina bark
1600‐1700	Quina‐quina bark powder is well‐spreading throughout Europe and Asia for febrile illness
1742	Quina‐quina tree is renamed as Cinchona tree by the botanist Carolus Linnaeus
1818	Quinine isolated from cinchona tree bark; found to be useful for the treatment of malaria
1894	Dr JF Payne's first description of the use of high doses of quinine to treat lupus.
1908	Quinoline nuclear structure is essential for antimalarial activity.
1920	Pamaquine is the first synthetic antimalarial drug
1930	Quinacrine is developed as an alternative to quinine to treat malaria
1931	Quinacrine is synthesized Ehrlich group and clinical trial
1934	Hans Andersag at Bayers Lab, synthesized Resochin by replacing the acridine ring of quinacrine with a quinoline ring
1939	Resochin is renamed as chloroquine; CQ is seemed too toxic for human use
1940	Quinacrine is used in Russia for lupus
World War II	British physicians noted soldiers who had inflammatory diseases improved on quinacrine
1945	HCQ is synthesized, less toxic than CQ in animal models. Clinical trials in USA approved for human use
1946	FDA‐approved CQ for treatment of malaria
1951	Remarkable effects of quinacrine in the treatment of lupus
1955	Plaquenil (hydroxychloroquine sulphate) is FDA‐approved to treat SLE and CLE lupus.
1956	CQ improves inflammation in RA
1959	Triquin (HCQ, chloroquine and quinacrine combination) is FDA‐approved to treat lupus
1960	CQ shows anticancer properties
1970	As a lysosomotropic agent, CQ is first shown to inhibit cell growth of tumour in vitro, as indicated by the accumulation of autophagic vacuoles.
The early 1970s	Banned clioquinol in response to controversy association with subacute myelo‐optic neuropathy (SMON) in Japan
1972	FDA‐approved for Triquin withdrawn and is pulled off the market
1974	CQ withdrawn from Japanese market because of mistaken claim as subacute myelo‐optico‐neuropathy (SMON) and retinopathy due to improper use with poor safety management
1980‐90	CQ analogs are investigated as autophagy inhibitors in vitro
1989	The first observation that CQ has an anticancer effect in Burkitt's lymphoma when CQ was given as prophylaxis against malaria in Tanzania
1998	The first study to observe CQ as autophagy inhibitor; the link between accumulation of cellular proteins and the inhibition of lysosomal degradation
2000	HCQ shows anticancer properties
2003	First clinical trial to evaluate the antitumour effects of CQ and found that CQ improved clinical outcome with autophagy inhibition in glioblastoma.
2007	In combination with anticancer drugs, CQ has a synergistic effect with other anticancer drugs
2009	HCQ is launching in Japan for clinical care
2010‐	CQ analogs and current research: bone diseases, cancers, hyperglycaemia, emerging viral infectious diseases (AIDS, SARS, dengue)
2014‐	HCQ in clinical trials: Multiple groups published results from phase I/II clinical trials using HCQ to selectively target autophagy in cancer patients
2017‐	CQ overcome resistance: Autophagy inhibition can overcome resistance to kinase inhibitors in tumour cells and in patients
2018‐2020	Microencapsulated CQ analogues for targeting CSCs^50^, ^66^

Note

Major references^16^, ^19^, ^59^, ^65^, ^71^

## 3 ROLE OF MITOPHAGY IN CANCER

Mitophagy (mitochondrial autophagy) is the selective identification, degradation and removal of spoiled mitochondria at the autophagolysosome.^73^ Mitophagy definitely varies from non‐selective bulk autophagy due to its selectivity and regulation of the autophagic cargo.^74^ Mitochondrial autophagy is co‐ordinately related to cellular homeostasis that responds to extracellular deviations (eg stress, energy, nutrients). On the one hand, autophagosome formation occurs at the junction of mitochondria with endoplasmic reticulum upon the stimulation of autophagy initiation. In this process, mitochondria participate from the outer mitochondrial membrane lipids to nascent isolation membrane of autophagosomes.^75^, ^76^ On the other hand, autophagy donates mitochondria maintenance by regulation of mitochondrial integrity, which may also be related to regulatory higher living processes.^77^

Mitophagy is triggered by stresses, DNA damage, inflammation, etc, and is an important mechanism for quality control of cellular bioenergetics and homeostasis by preserving mitochondrial integrity and actions.^78^ Any imperfections in mitophagy lead to mitochondrial dysregulation that changes metabolic pathways and alters cell fate which in turn initiates the incidence and aetiology of diseases, including cancer.^79^, ^80^, ^81^ Thus, both non‐selective bulk autophagy and selective mitophagy are impacted during tumorigenesis. Based on the type and stage context of the tumour, mitophagy may act either tumour‐promoting or tumour‐suppressive action.^80^, ^82^ Knockout of the vital regulatory mitophagy gene *PARK2* has been associated with several dissimilar human tumours, for example, TNBC.^83^ In addition, spontaneous hepatic tumour develops in mitophagy gene *Parkin* knockout mice which support mitophagy as a tumour‐suppressive mechanism.^82^ On the other hand, established tumours have been anticipated to employ mitophagy for supporting tumour growth under stress conditions.^80^

During initiation of tumour, mitochondria perform a main role in supplying nutrients essential for boosted cell propagation and angiogenesis.^74^ In addition, mitochondria contribute several events of cancers such as apoptosis resistance, oncogene‐associated transformation, reprogramming of metabolism, translation of protein, stemness of cancer, malicious repopulation and drug resistance.^84^, ^85^, ^86^ These solid foundation and proof‐of‐concept results strongly support the fact that mitochondria act as a fundamental metabolic centre vital for tumorigenesis. Thus, mitophagy mechanisms such as bioenergetics, biogenesis and cellular transductions of tumorigenesis have drawn the great attention for designing superb anticancer therapeutics.

### 3.1 Targeting mitophagy by antibiotics

Recent evidence suggests that CSCs are reliant on mitophagy pathways for their proliferation and clonal development, and pharmacological inhibition of mitochondrial biogenesis may effectively block CSCs.^9^, ^10^, ^87^, ^88^, ^89^ It is evident that various FDA‐approved agents particularly antibiotics modulate mitochondrial protein synthesis in mammalian cells as off‐target effects from its original antimicrobial use.^90^, ^91^, ^92^

Based on in vitro substitute CSC assays, numerous classes of FDA‐approved antibiotics including erythromycins (azithromycin), glycylcycline (tigecycline), tetracyclines (doxycycline), fluoroquinolones (levofloxacin and ciprofloxacin) and atovaquone (chloroquine analogues) have been found to markedly reduce tumorsphere development in several cancer cells including breast, lung, prostate and PDAC.^92^, ^93^ For instance, tigecycline selectively kills CSCs of acute myeloid leukaemia (AML) by suppression of mitochondrial translation.^94^

Azithromycin in combination with chemotherapy (paclitaxel and cisplatin) shows a positive response of one‐year survival of stage III/IV non‐small cell lung cancer (NSCLC) patients.^95^ Salinomycin selectively inhibits CSCs by impairing mitochondrial bioenergetic performance.^96^ Atovaquone performs as an oxidative phosphorylation (OXPHOS) inhibitor and significantly inhibits sphere formation in breast and colorectal CSCs without affecting normal fibroblasts.^97^ Pyrvinium pamoate, an anti‐parasitic agent, behaves as an OXPHOS inhibitor aiming mitochondrial complex II and competently stops mammosphere production.^98^ Doxycycline binds preferentially to the small subunit 28S ribosomes in mitochondria and erythromycin metabolites or chloramphenicol specifically fix to the mitochondrial ribosome large subunit 39S, thereby blocking biogenesis of mitochondria and thereby preventing protein translation as well as sufficient reduction in mammosphere production and bonafide CSC markers.^98^ Thus, it is interesting that FDA‐approved antibiotic‐mediated mitochondria targeting may contribute to eradicate cancer cells particularly CSCs and the anticancer efficacies of the antibiotics (Table 2).

**Table 2:** Major drugs targeting the lysosome in autophagy and mitophagy in cancer

Agent	Derivative	Water solubility	BBB permeability	Autophagy‐related mechanism of action	Target stage of autophagy	Therapeutic uses	Comments
Fusion and cargo degradation stages of autophagy							
Chloroquine	Aminoquinolines	Soluble	Permeant	Inhibition of lysosomal acidification	Fusion and degradation	Approved for malaria	Non‐specific inhibition of lysosomal functions
Hydroxychloroquine	Aminoquinolines	Soluble	Permeant	Inhibition of lysosomal acidification	Fusion and degradation	Approved for malaria, SLE and RA	Non‐specific inhibition of lysosomal functions
Quinacrine	Acridine	Soluble	Permeant	Inhibition of lysosomal acidification	Fusion and degradation	Accepted not established female sterility	Non‐specific inhibition of lysosomal functions
Mefloquine	Quinoline	Soluble	Permeant	Inhibition of lysosomal acidification	Fusion and degradation	Approved for malaria	Non‐specific inhibition of lysosomal functions
Quinine	Quinoline	Soluble	Permeant	Inhibition of lysosomal acidification; K^+^_ATP_ channel blockers	Fusion and degradation	Approved for malaria	Non‐specific inhibition of lysosomal functions
Lys05	Aminoquinolines	Soluble	Unknown	Inhibition of lysosomal acidification	Fusion and degradation	Pre‐clinical, cancer	Non‐specific inhibition of lysosomal functions
ARN16090	ARN5187 analog	Insoluble	Unknown	Inhibition of lysosomal acidification	Fusion and degradation	Pre‐clinical cancer	Also inhibition of NR1D2/REV‐ERBβ
VATG‐027	1,2,3,4tetrahydroacridine	Insoluble	Unknown	Inhibition of lysosomal acidification	Fusion and degradation	Pre‐clinical cancer	More potent autophagy inhibition than CQ
Clioquinol ionophore	8‐hydroxyquinoline	Insoluble	Unknown	Inhibition of lysosomal acidification	Fusion and degradation	Approved skin and urinary infections	Autophagy induction by disruption catalytic activity of mTOR
Bafilomycin A1	Macrolide antibiotic	Insoluble	Permeant	Lysosomal V‐ATPase inhibition	Fusion and degradation	Experimental agent	Universal V‐ATPase inhibitor (eg osteoclast, cancers)
Concanamycin A	Plecomacrolide antibiotics	Insoluble	Permeant	Lysosomal V‐ATPase inhibition	Fusion and degradation	Pre‐clinical for cancer	Universal inhibitor (eg osteoclast)
Archazolid	‐	Soluble	Unknown	Lysosomal V‐ATPase inhibition	Fusion and degradation	In vitro studies; a myxobacterial agent	Reduction in cathepsin B activity
Doxorubicin (Adriamycin)	Anthracycline antibiotic	Soluble	Impermeable	Lysosomal V‐ATPase suppression	Fusion and degradation	Approved for leukaemias, Hodgkin's lymphoma	Universal V‐ATPase inhibitor
Manzamine A	Manzamine alkaloid	Soluble	Unknown	Lysosomal V‐ATPase inhibition	Fusion and degradation	Pre‐clinical	v‐ATPase inhibition is similar to bafilomycin A
Cleistanthin‐A	Diphyllin glycoside	Soluble	Unknown	Lysosomal V‐ATPase inhibition	Fusion and degradation	In vitro studies	‐
Pepstatin A	Hexapeptide metabolite	Insoluble	Unknown	Lysosomal Aspartyl protease inhibitor	Partial Degradation (lysosomal proteolysis)	Not registered	A reversible non‐specific inhibitor
Leupeptin	Peptide antibiotic	Soluble	Permeant	Lysosomal protease and Ca^2+^‐dependent calpain inhibitor	Partial degradation (lysosomal proteolysis)	Not registered	A reversible non‐specific inhibitor
E64d	Fungal metabolite	Insoluble	Unknown	Lysosomal cysteine protease inhibitor	Partial degradation (lysosomal proteolysis)	Not registered	An irreversible non‐specific inhibitor
Elaiophylin	Macrodiolide antibiotic	Poorly *soluble*	Unknown	Abrogation of maturation of cathepsin B and D.	Degradation	Antibacterial and anthelminthic activities	Promotion of autophagosome accumulation
Nucleation and elongation stages of autophagy							
Spautin‐1	MBCQ	Insoluble	Unknown	Inhibition of USP10 and USP13 that target deubiquitination of Beclin 1	Nucleation	Pre‐clinical cancers	Inhibition of autophagy in a Beclin‐1‐independent manner
SAR405	‐	Insoluble	Unknown	Inhibition of Vps34	Nucleation	Pre‐clinical cancer	High protein and lipid kinase selectivity profile
Verteporfin	Benzoporphyrin	Insoluble	Permeant	Inhibition of LC3 lipidation	Elongation	Approved as PDT for macular degeneration and histoplasmosis	Autophagy inhibition independently of light

## 4 TARGETING LYSOSOME IN AUTOPHAGY AND MITOPHAGY BY CHLOROQUINE ANALOGUE AND ANTIBIOTICS

It has been found that CQ analogues at low concentration suppress bone resorptive activity of osteoclasts without affecting bone‐forming cells,^99^ and subtherapeutic antibiotic treatment (STAT) causes an increase in bone mineral density.^100^ Thus, combination of chloroquine analogues and mitochondrial‐targeted agents in subtherapeutic level (at low concentration) would be more therapeutic potentials against CSC‐related cancers and revolutionize the cancer research field without affecting normal cells.

## 5 CONCLUSIONS AND FUTURE PERSPECTIVE

According to the vast evidence on in vitro and mammalian/ animal models, it is expected to find positive impacts of the combination as a cancer therapy by manipulating the capacity of lysosome in autophagy‐mitophagy process in CSCs. Also, it is expected to discover the molecular mechanisms of therapeutic pathways in inhibition of CSCs without affecting severely in vital organs. Further studies are required at the subcellular levels of cancers for saving global people. At present, several non‐selective bulk autophagy inhibitors and selective mitophagy suppressors undertake in clinical trials (phases I and II) in which these agents are exploited together with a diversity of chemotherapeutic drugs in cancer treatments (Table 3). It is predicted that such type of combinatory autophagy inhibitors with understanding of the molecular regulatory mechanism of the autophagy will direct to certain revolution in the treatment of multiple human diseases including cancer in the near future.

**Table 3:** Ongoing clinical studies using the autophagy inhibitors CQ analogs in cancer treatment

Treatment strategy		Disease	Trial phase/ Status	Primary end‐point	Identifier	Sponsor
Inhibitor	Other agents					
CQ (Aralen)	None	Lung cancer	1/C	Safety	NCT00969306	Maastricht Radiation Oncology
		Breast cancer	2/R	Safety	NCT02333890	Ottawa Hospital Research Institute
	Carboplatin + gemcitabine	Malignant neoplasm	1/R	Safety	NCT02071537	University of Cincinnati
	Taxane	Breast neoplasms	2/R	Safety	NCT01446016	The Methodist Hospital System
HCQ (Plaquenil)	‐	Solid tumour	1/R	Safety	NCT03015324	University of Kentucky
		Solid tumour	1/R	Safety	NCT02232243	University of Kentucky
		Hepatocellular carcinoma	1 /2/R	Safety	NCT02013778	University of Pennsylvania
	Itraconazole	Ovarian cancer	1/2/R	Safety	NCT03081702	University Health Network
	Mitoxantrone + etoposide	Leukaemia, acute myelogenous	1/R	Safety	NCT02631252	University of Pittsburgh
	IL‐2	Metastatic renal cell carcinoma	1/2/R	Safety	NCT01550367	University of Pittsburgh
	Vorinostat	Malignant solid tumour	1/R	Safety	NCT01023737	Merck Sharp & Dohme Corp.
	Vorinostat + regorafenib	Colorectal cancer	2/R	Safety	NCT02316340	The University of Texas Health Science
	Gemcitabine + Nab‐paclitaxel	Pancreatic cancer resectable	2/R	Safety	NCT03344172	Pfizer, NCI
	Gemcitabine	Metastatic adenocarcinoma	1/2/R	Safety	NCT01506973	University of Pennsylvania
	Trametinib	Advanced BRAF mutant melanoma	1/2/R	Safety	NCT02257424	University of Pennsylvania
	Everolimus	Breast cancer stage IIB	2/R	Safety	NCT03032406	University of Pennsylvania
	Gemcitabine + carboplatin+ +etoposide	Small cell lung cancer	2/R	Safety	NCT02722369	University College, London
					
QC	Capecitabine	Colorectal adenocarcinoma	1 /2 ANT	Safety	NCT01844076	Milton S. Hershey Medical Center
	‐	Prostatic cancer	2/C	Safety	NCT00417274	Cleveland BioLabs
	Erlotinib	Recurrent NSCLC	1/C	Safety	NCT01839955	Case Comprehensive Cancer Center
VP	‐	Recurrent prostate cancer	1/R	Safety	NCT03067051	Princess Margaret Cancer Centre
	‐	Breast neoplasms	I/II/R	Safety	NCT02872064	University College, London
	‐	Pancreatic cancer	2/R	Safety	NCT03033225	Mayo Clinic
	Cisplatin	Pleural effusion, malignant	1/R	Safety	NCT02702700	Centre Hospitalier Universitaire
CLQ	‐	Leukaemia lymphoma, myeloma	1/T	Safety	NCT00963495	University Health Network
CM	Docetaxel, cabazitaxel	Prostate cancer	1/R		NCT03043989	Sidney Kimmel Comprehensive Cancer Center
	Lenalidomide	Lymphoma	2/R		NCT03031483	IELSG
	Dexamethasone + ixazomib +pomalidomide	Myeloma	1/2/R	Safety	NCT02542657	University of California
	Lenalidomide + dexamethasone	Relapse multiple myeloma	2/R	Safety	NCT02986451	Sun Yat‐sen University
	Thalidomide + cyclophosphamide +dexamethasone	Multiple myeloma	3/R	Safety	NCT02248428	Jinling Hospital, China
	Pioglitazone nivolumab treosulfan	Lung cancer, NSCLC	2/R	Safety	NCT02852083	University Hospital Regensburg
	Lenalidomide dexamethasone	Multiple myeloma	3/R	Safety	NCT02516696	Weill Medical College of Cornell University
CF	Neupogen	Early‐stage breast cancer	4/R	Safety	NCT02816112	Ottawa Hospital Research Institute
Col	‐	Hepatocellular carcinoma metastasis invasion	2/R		NCT01935700	Kaohsiung Medical University Chung‐Ho Memorial Hospital

Abbreviations

ANT, Active, not recruiting; C, completed; CF, ciprofloxacin; CF, ciprofloxacin; CLQ, clioquinol; CM, clarithromycin; CM, clarithromycin; Col, colchicine**;** CQ, chloroquine; Dox, doxorubicin; HCQ, hydroxychloroquine; LT, lucanthone; MBCQ, [4‐((3,4‐methylenedioxybenzyl)amino)‐6‐chloroquinazoline]; NCI, national cancer institute; NSCLC, non‐small cell lung cancer; QC, quinacrine; R, recruitment; S, suspended; T, terminated; VP, verteporfin.

## CONFLICT OF INTEREST

The author declares that there are no known competing financial interests or personal relationship that could have appeared to influence the work reported in this paper.

## AUTHOR CONTRIBUTION

**Md. Abdul Alim Al‐Bari:** Writing‐review & editing (equal).
